# Extra Supplement.—The Nursing Mirror

**Published:** 1888-10-13

**Authors:** 


### The Nursing Mirror.

All Contributions for this Supplement should be addressed "The Nursing Mirror," care of The Editor, 38, Old Jewry, E.C.

### En Passant.

FRENCH SISTERS OF CHARITY.—The poor Sisters who have been ejected from the Parisian hospitals they nursed so long, are accused of not keeping up with the times, falling behind in nursing, and thus falling into disrepute. Strange then that out of the thirty-four women who have received the ribbon of the Legion of Honour, twenty should be Sisters of Charity, and seven were decorated for service on the battlefield.

A NURSES' HOME FOR SUNDERLAND.—At a meeting held last month it was proposed to form a nurses' home at Sunderland for the supply of trained nurses to that town. It was also proposed to offer Miss Gregory, the head nurse of the Newcastle Home, the post of matron at Sunderland, and let her bring six of the Newcastle nurses with her. A circular has been issued to the public explaining the object of the movement and inviting subscriptions.

HANDBOOKS ON NURSING.—Miss E. A. Barnett is going to multiply the number of handbooks on nursing which already exist, by publishing her lectures delivered last year for the National Health Society. Miss Homersham, of the same society, has just issued a similar book. It certainly seems a pity that these two ladies did not combine to publish one book between them. The chief point of Miss Barnett's lectures was the last one on "Baby Nursing," with a demonstration to follow. There were some clever hints in the third, fourth, and fifth lectures, but the first two on "Nursing as a Profession" and "Nursing as a Fine Art," had all been said before a hundred times.

AN INDIAN INCIDENT.—Dr. Mackenzie has not been married twenty-five years for nothing. At the opening of the Dufferin Hospital at Nagpur, when a number of native ladies were present behind a screen, he made a direct appeal to them for help. These ladies were much astonished at being invited to take a part in forwarding a public undertaking, and before they could recover, Dr. Mackenzie adroitly contrived to make it a matter in which their claim to personal charms was more or less involved. If the husbands tried to raise difficulties he was sure, he said, they would know how to get over them. "The meanest curmudgeon would melt when deified by the lips of the woman he loved."

YELLOW FEVER IN FLORIDA.—Miss Delano, who was for many years a head nurse at Bellevue Hospital, New York, but who has lately been engaged in private nursing, offered her services to Dr. Mitchell soon after the yellow fever broke out, and is now working hard at Jacksonville. The New York papers call her "Our Florence Nightingale." A society of women, called the "King's Daughters," of Greenwich, Conn., have sent a lot of bedding to the yellow fever sufferers. On September 26th a concert was given in the Knickerbocker Conservatory, New York, for the sick at Jacksonville. Among the performers was Miss Maimie Horton, the brilliant whistler, a sister of Mrs. Shaw. As in the epidemic of some years ago, physician and nurses are outdoing the valour of battle in their quiet, self-commissioned martyrdoms. A writer in the *Globe* on this subject, says: "Heroic sisterhoods whose ranks closed in again and again to fill the gaps from voluntary life-surrender. Some of them, having made it a point of conscience to enter and search through every apparently tenantless house, in all the desolate streets which stood to mark that awful visitation—meeting many a ghastly sight and burying many a forgotten body."

PROPER TRAINING.—The Court of Governors of the Devon and Exeter Hospital have decided to discontinue the employment of nurses not residing in the building, and to employ a lady superintendent at a salary not exceeding £60 per annum, to supervise the nurses and nursing. The board also intends to build a home for the nurses and start a training school on an approved system. This is the second new nursing home which we announce this week, and which makes it evident that everywhere the subject of nursing is receiving greater consideration. Soon there will be no spot left in England where the old style of nurse is to be found: she will be swept off the face of the earth, and in her stead there will be known far and wide the trained, disciplined woman of to-day. "The provinces" is sometimes used as a term of contempt by Londoners, but with regard to nursing this can no longer be so, for some of the best training schools are now to be found attached to county hospitals.

THE REGISTRATION OF MIDWIVES.—We would remind any of our readers who live in the neighbourhood of Oxford that a meeting will be held there on October 25th, to discuss the question of the registration of midwives. Tickets and particulars can be had from Miss Wilson, 45, Colville Gardens, Bayswater. The subject is one of great interest, and has been before the public for years; midwives occupy an intermediate position between general practitioners and nurses, and should the Bill pass Parliament and work satisfactorily, the question of the registration of nurses might be better considered. Every intelligent person, however, must recognise that nurse and midwife registration are very different things, and that only those who are intimately acquainted with midwives and midwifery in all its bearings ought to speak on the last subject. At an inquest held at Wimbledon on September 29th, on an infant, the doctor stated he found some gruel-like substance in the stomach. Mary Berkwell, of Mitcham, deposed that she was called in to attend the mother on September 26th, and found that the child had been recently born. She gave it a little gruel. It died the next morning. Yet this woman called herself a midwife.

RELIGION AND NURSING.—Florence Madden, a Roman Catholic, remonstrates because she cannot be received at St. Thomas's, where the patients are Protestant. The priest of Valencia Island remonstrates because his Catholic flock have had a Protestant nurse forced on them. What a pity that Miss Madden and Miss Fitzgibbons cannot quietly change places and stop a scandal, both with regard to religion and nursing? We had sent us an account of the squabble at Valencia, which represented the priest in such a strange light we felt obliged to write to him for further information. He has kindly replied, and once more stigmatised the accusation that he cursed hospital and nurse as a "monstrous and offensive falsehood." That Protestants, without bringing forward proof, should go on reiterating charges which are thus denied is a disgrace to our religion. The whole facts of the case are published in the *Cork Examiner* of August 24th, if any care to see them. For our part we shall say no more on this subject, merely quoting the last part of Father Casey's letter, with which we heartily agree: "I set out with this proposition, and I shall stand or fall by it: That in any hospital in which all, or the overwhelming majority, of the patients are Catholics, they are entitled by every right to demand a Catholic nurse. And if the position be reversed, and Protestants form the majority, let them by all means have a Protestant nurse. The right we demand for ourselves we are willing to see enjoyed by others—we claim nothing more; we shall take nothing less."

### Notes from Australia.

### THE ALFRED HOSPITAL.

The foundation stone of the Alfred Hospital, Melbourne, was laid by Prince Alfred when he visited the Colonies. It has a frontage to St. Kilda Road, and covers with its grounds about twenty acres. Walking through the grounds from Punt Road, I was impressed by its position, which is one of the most eligible in the suburbs of Melbourne, near enough to the sea to get its reviving air—far enough away from the shore to be sheltered from cold winds. It is built of red brick, and one has at once in looking at the building a sense of strength, airiness, and utility. There are two wings, with a long building between. On the ground floor there is a good outer corridor, and on the upper floor is a most useful balcony the length of the said corridor. Both corridor and balcony are sheltered from north wind, which is the obnoxious wind in Melbourne, and they are easily accessible from the large wards. There are smaller balconies for the wings. For those who can go up and down stairs there are extensive grounds to walk in, or invalids can be taken up and down by a lift, which is so easy that a very slight nurse can work it comfortably. Outside the building there is a room or two for noisy patients—*i.e.*, for sleepless and delirious ones, who would otherwise disturb the patients in the large wards. Also in the gardens are some tents that are used when there is a pressure of fever patients. The two largest wards in the hospital have twenty-four beds in each; other wards vary in the number of beds, but all are in excellent order. In this hospital there are some patients who pay £2 10*s*. per week, and others, who have a room to themselves, pay £4 4*s*. per week. These rooms look just what should be for invalids: every comfort is provided, but there is no unnecessary encumbrance to the healthiness of the room. On the next floor above these wards is the room for the matron, who is a kind, strong, ladylike woman. She looks as if she had all the instincts of a nurse, a power of perpetual self-training for her work, and, above all, a great love for it, as well as plenty of tact with nurses and patients. She has forty-two nurses on her floor to superintend, and these all greatly respect her. The rooms for the nurses surprised me in one thing, *i.e.*, that they had pretty brackets, photographs, grasses, flowers, fans arranged round the walls, and in one case there were coloured muslin curtains to the window. "Oh, yes, we may have these if we like," I was told. This is less severe discipline than there would be in a hospital in London; but it seems to answer. The happy-looking woman who showed us round had been in the hospital three years and a-half, and one could only think, looking at her, that if one had to be ill it would be a pleasant fate to be nursed by her in the paying ward.

There are two sets of nurses for training—those who live always in the hospital, and those who are there from morning till five in the afternoon. All have the same opportunity for attending lectures for learning how to nurse.

The usefulness of the Alfred Hospital Nurses Training School is becoming more and more marked every year, and its popularity is increasing. There are 23 pupils on the roll, 12 of whom do not reside at the hospital, but contribute to their tuition, and eleven live in the institution, and give their services in return for the knowledge imparted to them. No fewer than 32 names had been put down as applicants for admission to the school, seven of them being from other colonies, but the want of accommodation rendered it impossible to receive any more than the number now on the roll. During the year 13 pupils passed their preliminary examinations, and eight have obtained the hospital's "Certificate of competency." Twenty nurses had been hired out during the year, and applications had been made for many more who could not possibly be spared owing to the demand within the hospital itself. A clinical school for the instruction and experience of medical students was opened in March last, and there are now 14 students attending the classes. Eight of the students are males and six females, but they all receive their instructions simultaneously, and, so far, the system has been found to work exceedingly well. The school is formally recognized by the council of the university, which has voted it a grant in aid for the present year of £100. The principal medical examining bodies in Great Britain have been asked to recognize the students' certificates, and there is every reason to believe that the request will be granted.

The following account of a visit to the Hospital for Sick Children, Melbourne, is taken from the *Argus*: Arrived at our destination, I was introduced to the kindly, genial matron, who, even in the midst of her morning duties, found time to accord me a smiling welcome, and to put me in charge of one of the nurses, who was directed to show me all that was to be seen. Visitors to the hospital are not merely tolerated, they are encouraged and made much of, for the committee, the doctors, and the matrons want to enlist the sympathy and help of the outside world for their little world of suffering children. They know well that one glance at the pale faces of these poor bairnies and at the tender care bestowed on them will do more to unloose purse-strings than the most pathetic appeal that was ever penned. "Because things seen are mightier than things heard." The evident good understanding which exists between the nurses and patients is testified by the smiles which light up the pale faces of the Maries and Johnnies, the Katies and Willies, when their kind nurse speaks to them. One merry little rascal, "Tommy," whose malady prevents his ever having learned to speak, although he is six years old, is not content with smiling, but laughs all over when Nurse Grace shows me his paralysed legs. He is quite ready for the game of romps which he expects to follow. And although this game only lasts a few minutes, and is played by a boy who cannot move the lower part of his body or say an articulate word, it is not at all devoid of fun. Among the patients, I saw one whose thin cheeks long weeks of sickness were powerless to blanch. This was "Wona," a little aboriginal. It was a curious sight to see this child of the native race surrounded by so many evidences of science and civilisation. One part of his savagery survives—he has a very strong objection to being bathed—a trait common to all children who have been brought up like savages, even should nature have given them fair skins. An Australian poet (Brunton Stephens) speaks of a like institution in an exquisite little poem, of which I borrow the concluding verses, which are an answer to the question, "Lord, when did we see thee hungry or thirsty, or a stranger?"—

"Inasmuch as though ye might not touch or tend them,
Ye were with them in your love to heal and save;
And were hand and feet to those who did befriend them,
By the gold and silver that ye gave.

"Find your treasure where your ransomed ones have hid it,
Take it back a thousandfold for your reward.
As ye did it unto them, to me ye did it;
Enter ye into the joy of your Lord."

### Appointment.

Victoria Home, Bournemouth.—Miss Bessie Noble has been appointed to the post of matron to the Victoria Home for Nurses. She was for three years at the Victoria Hospital, Chelsea, first as lady probationer and afterwards as sister of different wards. She was for a considerable time assistant superintendent of the Manchester Sick Poor and Private Nursing Institution.

### Notice.

Great Ormond Street Doll Show.—A special prize of a surgical case is offered for the best dressed doll in nursing uniform. A stall will be reserved for dolls dressed as nurses. All entries, with the fee of one shilling, must reach the Lady Superintendent by November 1st.

### Everybody's Opinion.

[*Correspondence on all subjects is invited. No communications can be entertained if the name and address of the correspondent is not given, or unless one side of the paper only be written on.*]

THE WHITECHAPEL MURDER.—I wish to thank you on behalf of myself and my fellow-nurse, who had to appear at the inquest on Annie Chapman, for your paragraph of September 22nd. I was away from the infirmary, and so escaped the ordeal of the inquest. It is true that we only carried out the orders given us, viz., to take the clothes off the body and wash it; but the *Globe's* remarks were the more uncalled for, as, before beginning, I asked the police inspector to tell us exactly what we were to do. He said that the doctor had seen the body, and that therefore we might wash it. (This he denied at the inquest.) The only direction he gave us was that the clothes were to be taken off without tearing or cutting. This we accomplished with great difficulty, owing to the terrible nature of the wounds. The chief rule of a hospital nurse is, or ought to be, implicit obedience, and I think in this case we have been blamed unjustly. I devoutly hope that we shall never in the course of our nursing experience see anything so terrible again. I know the sight has haunted both of us ever since, though we are neither of us nervous. We have, however, the satisfaction of knowing that the poor woman was decently arranged for her last rest, and that her clothes were not removed by the hands of male pauper officials.—THE SENIOR NURSE.

NURSES WHO ARE ROMAN CATHOLICS.—May I ask why you advise the public to refrain from giving any opinion on the subject of Miss Madden's treatment at St. Thomas's Hospital? I think it is precisely what is wanted—"Public Opinion" on what is already a sore subject amongst so many nurses. I have met with the same reception (more than once) as she did, since I became a Catholic. It is for the many influential personages who have recently so warmly espoused the Pension Fund for Nurses to consider the question why women should be debarred from obtaining the higher appointments of the nursing profession, or to be more correct, any post including that of probationer. It does not matter how good a nurse's testimonials are, and how conscientious and thorough in her duties she may be, as soon as the fact becomes known that she belongs to the Roman Catholic Church, the majority of those who elect the staff at the hospitals and infirmaries refuse to engage them. Why is it? Amongst those who have the interests of nurses at heart, surely there are some large-minded enough to point a way out of this difficulty. We have to attend Catholic patients, also others besides those belonging to the Church of England, and make not the slightest difference. Why, then, is the difference made between a Protestant and a Catholic nurse?—F. MANSON BRIDGE. [Our correspondent must remember that the founders of any institution have full power, and may reasonably be commended for establishing it upon principles they believe to be wisest and best. What hospital or institution, for instance, which is under the control of Roman Catholics employs any persons but Roman Catholics on its staff? It must further be borne in mind that it has been found in practice that the Roman Catholic clergy refuse to permit nurses who are members of their sect to comply with certain rules laid down by the anthorities for the good of their staff as a whole. We do not wish to enlarge upon this question, but a more liberal spirit must be shown by the authorities who control the action of individual Roman Catholics, and the management of Roman Catholic institutions, before the members of this faith can reasonably censure similar action on the part of others. This is probably not possible, however, and if so, the less said about the matter in question the better.—ED. *T. H.*]

THE QUESTION OF COURAGE.—With regard to the above question allow me to relate a little anecdote: A particularly vigorous speaker at a woman's rights meeting waving her long arms like the sails of a windmill, asked, "If the women of this country were to rise up in their thousands and march to the polls, I should like to know what there is on this earth that could stop them?" In the momentary silence which followed this peroration, a still small voice remarked: "A mouse!"—K. M.

TEACHERS OF CULTURE.—Permit me to call your attention to the literature recommended to nurses in a paper which professes to believe nurses to be a highly-educated set of women. The "Confessions of an Opium Eater," by *Dr.* Quincey, a biographical work called "Natural Law in the Spiritual World," and a book on nursing, called "The Mystery of Pain"! These are gross errors, but the sins of omission are even worse than those of commission. The list of books on nursing, which includes Mr. Hinton's metaphysical work, excludes Domville's "Manual" and Lückes "Lectures on Nursing." The list of poetry contains Archbishop Trench's poems and Whittier's "The King's Mission," but has no mention of Shakespeare, Milton, or the greatest poets. Surely I need say no more?—SISTER MARY.

### Vacancies.

The following vacancies are announced:—

**Matrons.**

Carnarvon Cottage Hospital.
Sheffield Infirmary; £70.

**Sister.**

Hospital for Women, Soho Square.

**Nurses.**

Royal Hospital for Women and Children.

Tunbridge Wells Hospital; £25.

Blackheath Institute; £20.

Nurses' Home, Norwich.

Birkdale Home, Southport.

Beckett Hospital; £25.

Stroud Hospital; £23.

Bolton Infirmary; £30.

New Hospital for Women; £25.

West Derby Union; £20.

Blackheath Institute for Trained Nurses; several.

Garrison Female Hospital, Woolwich; £19.

Bridgwater Infirmary; £22.

Nottingham Institution.

Kingston Home; Hull; several.

St. Saviour's Infirmary; £20.

Victoria Home, Bournemouth.

Southport Nurses' Home.

Royal Albert Hospital; £25.

Mr. D. E. Wilson, of 96, 97, 98*a*, Wimpole Street, London, W., has several vacancies for Male and Female Nurses.

Home for Trained Nurses, Bath; £25.

Philanthropic Society's Farm School, Redhill; £25.

County of Cornwall Trained Nurses' Home, Truro; two.

**District Nurses.**

Disley; £70. Dublin. Egham; £50.

**Probationers.**

Lancaster Infirmary; £13. Grantham Hospital.

### Examination Questions.

[Copies of similar questions will be gladly received by the EDITOR.]

With regard to the training of volunteer nurses, the following questions are suggested in Surgeon-Major Evatt's paper on *Common Accidents*:—

(1) What is a bearer Company?
(2) What are the equipments of a field hospital, and how many men should it hold?
(3) What is the function of a regimental ambulance detachment?
(4) What are the étappen posts?
(5) What is the base of operations?
(6) Trace the course of a wounded soldier from the time he is hit in battle until he reaches England.
(7) Where are female nurses employed in war?

### Notes and Queries.

TO CORRESPONDENTS.—1. Questions may be written on post-cards. 2. Advertisements in disguise are inadmissible. 3. In answering a query please quote the number. 4. If a private answer is desired, a stamped addressed envelope must be forwarded.

**Queries.**

(69) *The Rotunda Hospital.*—Will any nurse who has been trained in midwifery at the Rotunda Hospital, Dublin, as resident pupil, kindly give me the benefit of her experience. Is the work heavy, and are the hours long? Is there much to do besides nursing and the lectures? Are the home arrangements comfortable? Do the pupils have separate bed-rooms or cubicles?—*S. M. W.*

(70) *Nursing Home.*—Can you tell me of a home where a lady of some hospital experience will be received on the staff?—*Nurse H.*

(71). *The Red Cross.*—Will someone kindly tell me where a certificated nurse must apply to be received as a Red Cross nurse.—*Nurse J.*

**Answers.**

*M. G.*—The book is published in America and cannot be got in England. Get Domville's "Manual," or Luckes "Lectures on Nursing," both published by Messrs. Churchill, from any bookseller.

